# Effects of an unsupervised Nordic walking intervention on cognitive and physical function among older women engaging in volunteer activity

**DOI:** 10.1016/j.jesf.2021.06.002

**Published:** 2021-07-02

**Authors:** Yuta Nemoto, Ryota Sakurai, Susumu Ogawa, Kazushi Maruo, Yoshinori Fujiwara

**Affiliations:** a Research Team for Social Participation and Community Health, Tokyo Metropolitan Institute of Gerontology, 35-2 Sakae-cho, Itabashi, Tokyo, 173-0015, Japan; b Faculty of Medicine, University of Tsukuba, 1-1-1 Tennodai, Tsukuba Ibaraki, 305-8575, Japan

**Keywords:** Aging, Exercise, Functional mobility, Handgrip strength, Pole walking

## Abstract

**Background:**

Nordic walking (NW) has been reported as a safe and effective exercise mode. However, the effects of NW on cognitive function are unknown. This study examined the effects of an unsupervised NW intervention on cognitive and physical function among older women engaging in volunteering.

**Methods:**

Forty-seven women aged ≥70 years were enrolled and assigned into three groups (NW (n = 16); walking (n = 19); control group (n = 12)) based on residential areas. Participants in NW and walking groups received a pedometer and recorded daily step counts. The NW group received poles and 2 h of NW instruction. Participants were encouraged to perform the exercise individually more than once a week during the 3-month intervention. As baseline and follow-up assessments, cognitive function (Montreal Cognitive Assessment [MoCA-J] and Trail Making Test), physical function (handgrip strength, walking speed, balance ability, the Timed Up and Go test, and functional capacity), and objective physical activity were evaluated.

**Results:**

In the NW group, physical activity, maximal walking speed, and MoCA-J scores were improved during the intervention period. In the walking group, physical activity was increased after the intervention. Analysis of covariance showed that maximal walking speed among the NW group significantly improved compared with the walking group. Sub-group analysis of participants who exercised more than once a week showed that handgrip strength, gait speed, and MoCA-J scores were significantly improved in the NW compared with the walking group.

**Conclusion:**

NW intervention improved cognitive and physical function compared with simple walking among older women.

## Introduction

Long-term volunteering activities are associated with the maintenance of physical function, intellectual activity, and social networks,^1^^,^^2^ as well as the prevention of brain atrophy.^3^ Although older women are at greater risk than men for functional disability,^4^ they are less likely to engage in group activity long-term due to declining physical and cognitive function.^5^^,^^6^ In addition, older women have an increased risk of frailty and cognitive impairment.^7^ Thus, targeted programs for preventing functional decline among older women may help maintain long-term social engagement in this population.

Walking-based exercise contributes to maintaining physical function.^8^^,^^9^ However, older women with health problems are less likely to be active because of the risk of injury.^10^ Nordic walking (NW) is a safe and effective walking exercise performed using specially designed poles. The NW technique involves a backward pole position during the loading phase, control of the poles through the grip and straps, and the active and dynamic use of poles. The use of poles actively engages the upper-limb muscles to propel the body forward.^11^^,^^12^ NW can be performed in a wide range of locations, from parks and roads to hiking trails. NW has been found to increase oxygen consumption and energy expenditure, improve physical fitness, and reduce rates of falling among older adults, compared with walking without poles.^11^, ^12^, ^13^, ^14^ A previous study reported that 8 weeks of NW training enhanced walking economy by reducing co-contraction of the upper limb muscles.^15^ NW training improved optimal and self-selected walking speed and locomotor rehabilitation index among sedentary older adults.^16^

In addition, because NW exercise requires the coordination of the upper and lower limbs using poles, it may have similar cognitive effects to dual-task walking.^17^ The effects of simple walking on cognitive function are small compared with multicomponent exercise.^18^ These findings suggest that NW may be more effective for improving and maintaining cognitive and physical function. However, no previous studies have investigated the effects of NW on cognitive function compared with simple walking among healthy older women.

A previous meta-analysis reported that supervised exercise produced more substantial health effects than unsupervised programs.^19^ However, for community-dwelling populations, developing effective unsupervised programs may be beneficial because they require less staff and are less expensive than supervised interventions.

The current study aimed to evaluate the effectiveness of an unsupervised NW intervention on cognitive and physical function compared with simple walking among older women who engage in volunteering. We hypothesized that the effects of an unsupervised NW program on physical and cognitive function would be significantly greater than those in the walking and control groups.

## Methods

### Study design and participants

The present study was a preliminary intervention study examining the effects of a 3-month unsupervised NW program on cognitive and physical function among older women engaging in volunteer activity.

The study participants were selected from REPRINTS (REsearch of PRoductivity by INTergenerational Sympathy) groups in three urban areas in Japan, including the city of Kanagawa (Kawasaki) and two wards of Tokyo (Toshima and Suginami). The activities included picture-book reading to children in elementary schools, kindergartens, and public childcare centers once every 1–2 weeks.^1^^,^^20^ Among the volunteers, women aged ≥70 were recruited in July 2017. The exclusion criteria were as follows: having Parkinsonism or any neurological disorder involving a residual motor deficit (e.g., severe stroke); having active osteoarthritis affecting the lower limbs; using psychotropic medications that affect motor performance. Forty-seven older women who completed the baseline assessment were assigned to three groups (NW (n = 16), walking (n = 19), and control (n = 12) groups) based on residential areas.

Participants gave written informed consent at enrolment before assessment. The study protocol was approved by the Ethics Committee of the Tokyo Metropolitan Institute of Gerontology. The study protocol was registered in the UMIN-CTR (registration number: UMIN000029651).

### Intervention program

The intervention program was conducted for 3 months (September 2017 to December 2017) in each area.

All groups received a baseline health check-up with feedback and a 1-h health education lecture about the significance of physical activity for maintaining cognitive and physical function. Among the study participants, the amount of moderate to vigorous physical activity (MVPA) at baseline was high (mean: 156 min/week). The Japanese Sports Agency recommends performing exercise at least once a week. Thus, we encouraged participants to perform the exercise more than once a week, although 150 min/week of MVPA and multicomponent physical activity ≥3 days/week are recommended in physical activity guidelines.^21^

According to a previous study, self-monitoring using a pedometer can significantly increase physical activity.^22^ Thus, the NW and walking groups were given a pedometer and instructed to measure their daily step count.

Furthermore, the NW group participants received NW poles and a lesson about the appropriate NW technique for one session lasting 2 h. Qualified NW instructors provided the lecture. Participants were then asked to perform NW as part of their daily traveling, and to perform it more than once a week. Participants could consult with instructors about NW techniques whenever they wanted.

The exercise was unsupervised, and the location of exercise was not specified; participants could freely choose to engage in NW or exercise during the 3 months of the intervention period.

### Measurements

As baseline and follow-up assessments, cognitive function, physical function, and physical activity were evaluated. The staff member performing the assessments was not involved in implementing any aspect of the intervention and knew the participants only by their ID.

### Cognitive function

Cognitive function measurement comprised the Japanese version of the Montreal Cognitive Assessment (MoCA-J)^23^ and Trail Making Test (TMT) A and B.

MoCA-J is an assessment tool for general cognitive function, including attention, concentration, executive function, memory, language, visuoconstructional skills, conceptual thinking, calculations, and orientation. MoCA-J scores range from 0 to 30, and higher scores indicate better cognitive function. This measure has been reported to have high sensitivity and specificity for detecting mild cognitive impairment.^23^

In the TMT-A, participants were asked to connect the numbers (1–25), which were randomly scattered in ascending order. In TMT-B, participants were asked to connect the numbers (1–13) and Japanese Hiragana letters (12 letters) alternately. Participants performed both tests as quickly as possible, and administration time was measured. To measure executive control function, Delta TMT was calculated by subtracting the administration time of TMT-A from that of TMT-B.

### Physical function

Physical function assessment comprised handgrip strength, 5-m walk test, single-leg stance test, Timed Up and Go test, and functional capacity.

Handgrip strength was evaluated using a Smedley dynamometer. Dominant handgrip strength was measured twice, and the higher value was accepted. For the 5-m walk test, the start and endpoints of an 11-m walkway were marked, with additional marks at the 3- and 8-m lines. Walking speed was evaluated from the first footfall after the 3-m line to the first footfall after the 8-m line. Participants walked as fast as possible twice, and the faster time was recorded. In the single-leg stance test, which assesses balance ability, participants stood on one leg, keeping their eyes open and focusing on a mark at eye level. Administration time started from when the participant's leg left the floor. The test was stopped when the foot touched down or the performance time reached 60 s. In the Timed Up and Go test, participants in a seated position stand up and walk 3-m, turn around, walk back to the chair, and sit down as fast as possible. An observer measured the time taken by the participants to complete the procedure. The test was administered twice, and the faster time was recorded. Functional capacity was assessed using the Tokyo Metropolitan Institute of Gerontology Index of Competence score (TMIG-IC).^24^ The measure comprised 13 items, and scores ranged from 0 to 13. Higher scores indicated better physical function.

### Physical activity

The amount of MVPA was measured using an accelerometer (Lifecorder EX, Suzuken Co. Ltd., Nagoya, Japan). Participants wore the device on their waist for a week except during bathing, sleeping, and aquatic exercise. Following previous studies,^25^^,^^26^ data from participants who wore the device for at least 13 h a day for more than 4 days were included. The device records the activity intensity (level one to nine) every 4 s. The activities were classified into “light-intensity (<3.0 MET: level one to three)”, “moderate-intensity (3.0–6.0 MET: level four to six)”, and “vigorous-intensity (>6.0 MET: level seven to nine)”.^27^ The amount of MVPA (level four to nine) was calculated with the data from 0:00 to 23:59 excluding non-wearing time and was multiplied by seven to compute the weekly MVPA. Because we aimed to evaluate the performance time of exercises such as NW and simple walking, light-intensity, and sitting time were not assessed.

### Frequency of performing the exercise

At the follow-up assessment, participants in the NW group were asked about the frequency of performing NW during the intervention period, while participants in the walking and control groups were asked about the frequency of performing exercise. Those who performed exercise more than once a week were classified as “regular exercisers.”

### Statistical analysis

One-way analysis of variance and chi-square test were conducted to evaluate the group difference in baseline characteristics. The changes in each outcome measure in each group were evaluated with paired t-tests. Analysis of covariance (ANCOVA) was conducted to evaluate the effectiveness of the program because ANCOVA has more statistical power in intervention studies.^28^ The analysis models included the difference of least squares (LS) means of cognitive and physical functions between groups based on ANCOVA as dependent variables, group (NW, walking, and control) as an independent variable, and age, educational attainment, and the baseline value of each dependent variable as covariates. Tukey's correction was conducted for multiple comparisons. Because the participants were asked to perform NW or exercise more than once a week, subgroup analysis, which examines the effects of the program among individuals who adhered to the intervention, was conducted. The model included participants who performed NW more than once a week among the NW group and those who performed exercise more than once a week among the walking and control groups. Analysis using the same ANCOVA model was used for the main analysis.

For missing information on variables used in the study, we performed multiple imputations by chained equations, assuming missing at random. Twenty data sets were created, and combined results of each data set to obtain the estimates.

P < 0.05 was considered to indicate statistical significance, and SAS 9.4 (SAS Institute, Cary, NC) was used for all statistical analyses.

## Results

**Fig. 1:**
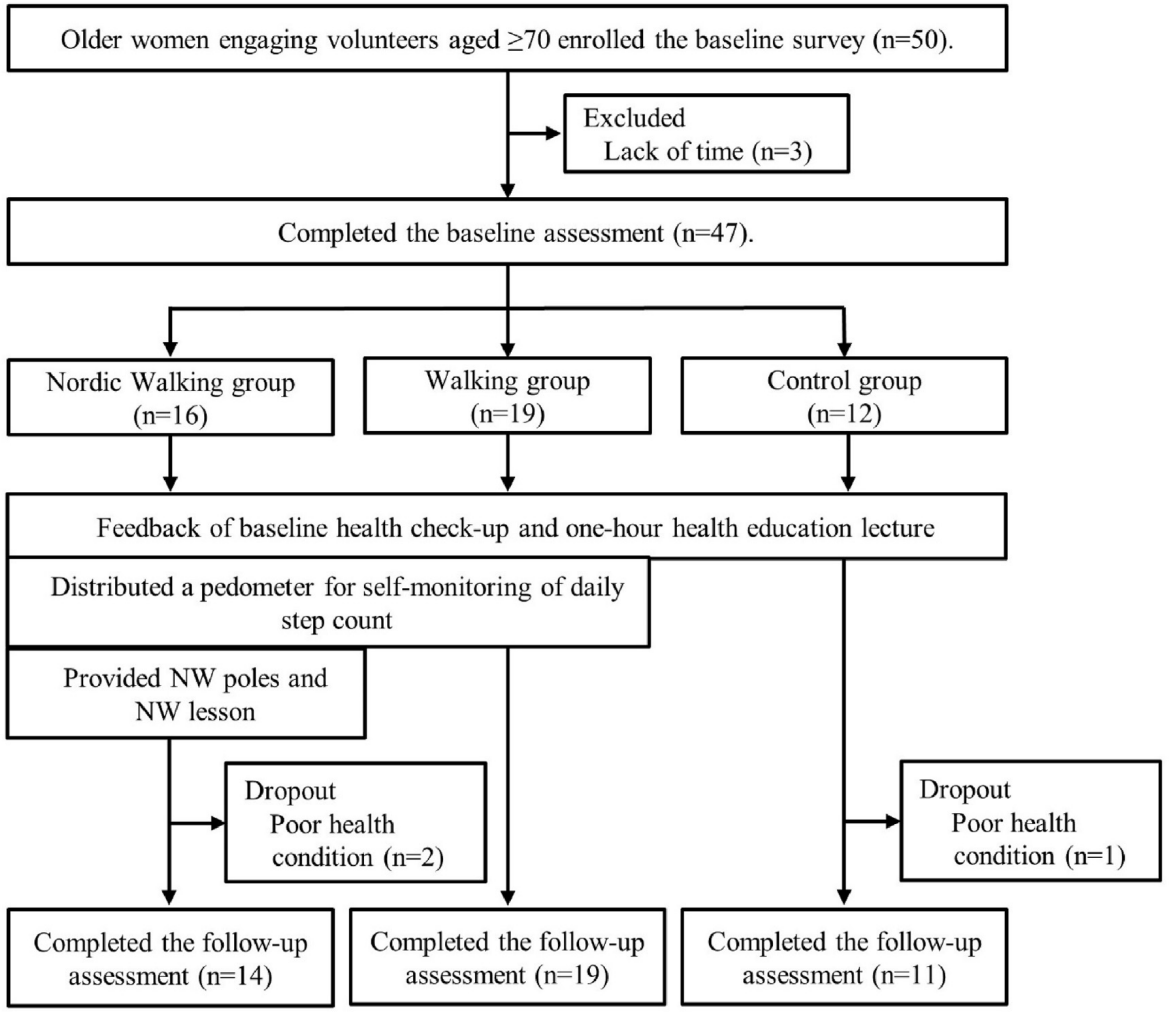
Flow diagram of this study.

Of 47 participants, three dropped out due to poor physical condition during the intervention period. Thus, 44 participants completed the intervention program and follow-up assessments (NW: n = 14, walking: n = 19, control: n = 11) (Fig. 1). No adverse events relating to the intervention were reported.

Baseline characteristics are shown in Table 1. There were no group differences in baseline characteristics. Participants exhibited high cognitive and physical function (TMIG-IC means: 12.2 [1.1] out of 13, MoCA-J score means: 26.1 [3.0] out of 30) (Table 1).

**Table 1:** Baseline characteristics of study participants.

	NW group (n = 14)		Walking group (n = 19)		Control group (n = 11)		Group difference
	Mean	SD	Mean	SD	Mean	SD	P-value
Age (year)	73.2	2.1	74.5	4.4	76.2	3.5	0.13
MVPA (min/week)	120.2	80.0	187.5	91.9	152.8	129.9	0.18
TMIG-IC score (point)	12.1	1.4	12.2	1.0	12.5	0.9	0.60
Dominant hand grip strength (kg)	22.4	5.0	22.6	4.4	22.4	4.6	0.99
Maximal walking speed (m/s)	2.0	0.3	2.0	0.5	2.0	0.4	1.00
Single leg stance test (s)	49.6	20.8	42.9	22.8	36.9	20.2	0.35
Timed Up and Go test (s)	5.0	0.8	5.9	1.3	5.4	0.9	0.07
MoCA-J score (point)	24.9	3.3	25.5	2.9	26.6	3.0	0.38
Delta TMT (s)	87.8	85.0	61.1	41.2	115.4	117.9	0.21
	**n**	**%**	**n**	**%**	**n**	**%**	**P-value**
Educational attainment (<13 years)	8	57.1	6	31.6	3	27.3	0.11
Mental health (poor)	1	7.1	6	31.6	3	27.3	0.19

**Notes:** Differences between groups in baseline characteristics were evaluated using one-way analysis of variance and chi-square test.

**Abbreviations:** NW; Nordic walking, SD; Standard deviation, MVPA; Moderate to vigorous physical activity, TMIG-IC; Tokyo Metropolitan Institute of Gerontology Index of Competence score, MoCA-J score; Montreal Cognitive Assessment score, TMT; Trail making test.

Table 2 shows the changes in cognitive and physical function during the program in each group. In the NW group, MVPA (Difference; 53.8 min/week, 95% CI; 19.9 to 81.7), maximal walking speed (Difference; 0.2 m/s, 95% CI; 0.0 to 0.4), and MoCA-J scores (Difference; 1.7 points, 95% CI; 0.4 to 3.0) were significantly improved. In the walking group, MVPA (Difference; 55.8 min/week, 95% CI; 10.1 to 101.4) was significantly increased. No significant differences were found in the control group (Table 2).

**Table 2:** Changes in outcome measures between pre- and post-evaluation.

	Nordic walking group (n = 14)				Walking group (n = 19)				Control group (n = 11)			
	Difference	95% Cl		P-value	Difference	95% Cl		P-value	Difference	95% Cl		P-value
MVPA (min/week)	53.8	19.9	87.7	<0.01	55.8	10.1	101.4	0.02	−0.9	−30.3	28.6	0.95
Dominant hand grip strength (kg)	−0.4	−2.2	1.3	0.60	−0.7	−1.6	0.2	0.10	−0.9	−2.4	0.7	0.25
Maximal walking speed (m/s)	0.2	0.0	0.4	0.04	0.0	−0.2	0.1	0.50	0.0	−0.1	0.1	0.54
Single leg stance test (s)	−0.4	−4.1	3.3	0.82	4.0	−0.4	8.4	0.08	7.5	−4.7	19.7	0.20
Timed Up and Go test (s)	0.0	−0.3	0.3	0.88	−0.1	−0.4	0.2	0.49	0.1	−0.2	0.4	0.42
MoCA-J score (point)	1.7	0.4	3.0	<0.01	0.8	−0.4	2.0	0.17	−0.2	−1.8	1.5	0.81
Delta TMT (s)	−26.0	−71.9	19.9	0.27	29.3	−14.2	72.8	0.17	−22.3	−97.3	52.7	0.56

**Notes:** Paired *t*-test was conducted. Difference indicates a difference of outcome measures between pre- and post-evaluation in each group.

**Abbreviations:** MVPA; Moderate to vigorous physical activity, MoCA-J score; Montreal Cognitive Assessment score, TMT; Trail making test.

The effects of the intervention on cognitive and physical function are shown in Table 3. ANCOVA revealed that participants in the NW group exhibited significantly greater improvement in maximal walking speed compared with the walking group (Difference; 0.2 m/s, 95% CI; 0.1 to 0.4) (Table 3).

**Table 3:** Comparison of the effects on physical and cognitive function between the three groups.

	NW - Walking				NW - Control				Walking - Control			
	Difference	95% Cl		Adjusted P-value	Difference	95% Cl		Adjusted P-value	Difference	95% Cl		Adjusted P-value
MVPA (min/week)	−2.7	−63.6	58.3	0.93	59.6	−9.7	128.9	0.09	62.3	2.6	122.0	0.04
Dominant hand grip strength (kg)	0.6	−1.0	2.2	0.48	1.0	−0.9	2.9	0.30	0.4	−1.6	2.4	0.86
Maximal walking speed (m/sec)	0.2	0.1	0.4	<0.01	0.1	−0.1	0.3	0.40	−0.1	−0.3	0.1	0.31
Single leg stance test (sec)	−4.4	−12.2	3.5	0.27	−7.6	−16.7	1.4	0.10	−3.3	−11.3	4.6	0.42
Timed Up and Go test (sec)	−0.01	−0.52	0.50	0.99	−0.06	−0.64	0.53	0.97	−0.05	−0.59	0.49	0.97
MoCA-J score (points)	0.6	−0.8	2.0	0.40	1.0	−0.7	2.7	0.25	0.4	−1.0	1.8	0.59
Delta TMT (sec)	−33.4	−82.9	16.2	0.19	−35.9	−93.8	22.0	0.22	−2.5	−55.4	50.4	0.93

**Notes:** Analysis of covariance (ANCOVA) was conducted. Age, educational attainment, and the baseline value of each dependent variable were adjusted. Difference indicates that the difference of least square means between groups based on ANCOVA. Adjusted P-values indicate the multiplicity-adjusted P-values based on Tukey's method.

**Abbreviations:** NW; Nordic walking group, MVPA; Moderate to vigorous physical activity, MoCA-J score; Montreal Cognitive Assessment score, TMT; Trail making test.

The results of the sub-group analysis are shown in Table 4. Both participants who performed NW more than once a week in the NW group and those who performed exercise more than once a week in the walking and control groups were included in the analysis (33 individuals, NW: n = 8, walking: n = 16, control: n = 9). The analysis revealed that grip strength (Difference; 2.0 kg, 95% CI; 0.1 to 0.4), maximal walking speed (Difference; 0.3 m/s, 95% CI; 0.1 to 0.5), and MoCA-J score (Difference; 1.7 points, 95% CI; 0.1 to 3.2) were significantly improved in the NW group (Table 4).

**Table 4:** Comparison of the effects on physical and cognitive function between the three groups (sub-group analysis).

	NW - Walking				NW - Control				Walking - Control			
	Difference	95% Cl		Adjusted P-value	Difference	95% Cl		Adjusted P-value	Difference	95% Cl		Adjusted P-value
MVPA (min/week)	14.3	−53.6	82.2	0.68	61.7	−16.3	139.7	0.12	47.4	−16.5	111.1	0.15
Dominant hand grip strength (kg)	2.0	0.2	3.9	0.03	2.0	−0.2	4.1	0.08	−0.1	−1.8	1.7	0.99
Maximal walking speed (m/sec)	0.3	0.1	0.6	<0.01	0.2	−0.1	0.5	0.23	−0.1	−0.4	0.1	0.41
Single leg stance test (sec)	−2.7	−12.5	7.1	0.59	−5.4	−16.9	6.0	0.35	−2.8	−12.2	6.7	0.57
Timed Up and Go test (sec)	0.10	−0.40	0.60	0.89	0.03	−0.51	0.57	0.99	−0.06	−0.53	0.41	0.95
MoCA-J score (points)	1.7	0.1	3.2	0.04	1.7	−0.2	3.5	0.08	0.0	−1.6	1.6	1.00
Delta TMT (sec)	−51.5	−108.1	5.2	0.08	−46.1	−115.3	23.2	0.19	5.4	−55.8	66.6	0.86

**Notes:** Analysis of covariance (ANCOVA) was conducted. The model included individuals in the NW group who performed NW more than once a week, and individuals in either the walking or control groups who performed any type of physical exercise more than once a week (NW: n = 8, Walking: n = 17, Control: n = 9). Age, educational attainment, and the baseline value of each dependent variable were adjusted. Difference indicates that the difference of least square means between groups based on ANCOVA. Adjusted P-values indicate multiplicity-adjusted P-values based on Tukey's method.

**Abbreviations:** MVPA; Moderate to vigorous physical activity, MoCA-J score; Montreal Cognitive Assessment score, TMT; Trail making test.

There were no differences between the results of the analysis using imputed data and those with complete case data.

## Discussion

This study examined the effects of the unsupervised NW intervention, revealing increased physical activity, walking speed, and MoCA-J scores in a pre-and-post comparison. In addition, the NW program improved gait speed compared with simple walking in ANCOVA. The sub-group analysis indicated that NW enhanced cognitive and physical function compared with simple walking among individuals who perform NW or exercise more than once a week. These results suggest that NW was more effective for maintaining physical and cognitive function than simple walking.

NW increased walking speed compared with simple walking, possibly reflecting an improvement of lower limb muscle function. Recent studies have suggested that using NW poles increases stride length and gait speed^29^ and strengthens the lower limb muscles,^13^^,^^30^ potentially resulting in increased maximal walking speed. Another possible reason is that NW may improve walking economy compared with simple walking. In older adults, increased joint stiffness increases lower limb muscle co-contraction to enhance stability. A previous study suggested that antagonist thigh muscle co-contraction during walking has a linear relationship with the cost of walking.^31^ NW training might reduce co-contraction from upper limb muscles, lowering the cost of walking and increasing walking speed.^15^^,^^16^

Sub-group analysis revealed that NW significantly improved handgrip strength, maximal walking speed, and general cognitive function. This finding suggests that regularly performing NW, but not intermittently performing NW, contributed to the improvement/maintenance of cognitive and physical function among older women. The improvement of grip strength in the NW group was consistent with previous studies.^32^^,^^33^ Because NW involves gripping poles and pushing them against the ground during walking, handgrip strength is expected to improve.

The NW group exhibited significantly improved MoCA-J scores, which is likely to be related to the characteristics of NW. NW may be considered a multicomponent exercise that comprises cardiovascular and coordination training.^34^ A previous study reported that cardiovascular and coordination training differentially affected cognitive function.^35^ Although both training types improve executive function and perceptual speed, cardiovascular training was associated with increased sensorimotor network activation, whereas coordination training was associated with increased visual-spatial network activation.^35^ NW may affect both the sensorimotor and visual-spatial networks. Furthermore, NW involves coordinating the upper and lower limbs using poles, with similarities to dual-task walking involving walking while performing a cognitively demanding task. A previous study of older Japanese adults with mild cognitive impairment reported dual-task walking induced prefrontal activation and improved cognitive function.^36^ Importantly, a close relationship has been suggested between dual-tasking ability and cognitive impairment, including Alzheimer's disease pathology.^37^ Performing NW involves the control of poles during waking, which might serve as a cognitive task.

The unsupervised NW program might be suitable for community-dwelling older adults to maintain physical and cognitive function. Unlike supervised programs that control exercise dosage, older adults in an unsupervised NW program can decide the intensity, frequency, and duration depending on their health condition. In terms of equivalent perceived exertion, heart rate and oxygen consumption during NW are higher than walking without poles.^11^ Thus, the intensity and duration of NW may be higher in unsupervised training than in supervised programs among healthy older women. Furthermore, individuals with chronic low back pain can perform NW, which reduces the risk of pain-related difficulties. Using poles during uphill walking decreases contraction of the erector spinae muscle and prevents muscle overuse. These findings indicate that NW may reduce the effort required to control trunk oscillations and work production during NW.^11^

Although there were substantial changes in gait speed and MoCA-J scores between pre-and-post assessments in the NW group, but not in the control group, significant group differences in cognitive or physical function change were not detected in multivariate analyses. The statistical models had low statistical power because of the small sample size. Additionally, because we did not restrict and monitor participants’ physical activity during the intervention, it is possible that participants in the control group performed frequent exercise or volunteering activity during the intervention and maintained better physical and cognitive function than expected.

The strength of this study is that we identified the effects of an unsupervised NW program on cognitive function compared with simple walking among healthy older women. Unsupervised programs are beneficial for community-dwelling healthy older adults. Although there is a need to further examine the effects of this intervention in randomized controlled trials, the current findings suggest that women-oriented unsupervised programs could be disseminated in community settings. However, this study involved several limitations that should be considered. First, the sample size was insufficient for detecting the effects of the program, and this issue also limited the generalizability of the current results. Second, participants’ high cognitive and physical function at baseline and the relatively short intervention period may have limited the effectiveness of the program. Third, we used the MoCA-J to measure cognitive changes; however, there might be ceiling effects among study participants with high cognitive function. Additional cognitive testing should be conducted to detect the effects of NW among healthy older adults. Fourth, although multiple comparisons were performed, multiplicity adjustment may have been insufficient because many dependent variables were examined.

In conclusion, this study examined the effects of unsupervised NW intervention on cognitive and physical function compared with simple walking among older women engaging in volunteer activity. Our preliminary findings revealed that an unsupervised NW program was associated with improved walking speed. Moreover, performing NW more than once a week improved handgrip strength, walking speed, and cognitive function compared with simple walking. Our findings indicated that NW improved cognitive and physical function, which may support long-term volunteer activity, among older women engaging in volunteer activity.

## Funding

This study was supported by research funding from Unicharm Corporation, and SINANO Co., Ltd. These funders had no role in study design, data collection, data analysis, or preparation of the manuscript.

## Declaration of competing interest

YF received research grants from Unicharm Corporation.

## Authors statement

Study conception and design: YN, RS, SO, YF

Acquisition of data: YN, RS, SO, YF

Analysis and interpretation of the data: YN, RS, KM, YF

Drafting the paper: YN, RS

Critical revision: RS, SO, KM, YF

All authors read and approved the final version of the manuscript.
